# Dimensional crossover in a layered ferromagnet detected by spin correlation driven distortions

**DOI:** 10.1038/s41467-019-09663-3

**Published:** 2019-04-10

**Authors:** A. Ron, E. Zoghlin, L. Balents, S. D. Wilson, D. Hsieh

**Affiliations:** 1 Department of Physics, California Institute of Technology, Pasadena, CA 91125 USA; 2 Institute for Quantum Information and Matter, California Institute of Technology, Pasadena, CA 91125 USA; 3 Materials Department, University of California, Santa Barbara, CA 93106 USA; 4 Kavli Institute for Theoretical Physics, University of California, Santa Barbara, CA 93106 USA

## Abstract

Magneto-elastic distortions are commonly detected across magnetic long-range ordering (LRO) transitions. In principle, they are also induced by the magnetic short-range ordering (SRO) that precedes a LRO transition, which contains information about short-range correlations and energetics that are essential for understanding how LRO is established. However these distortions are difficult to resolve because the associated atomic displacements are exceedingly small and do not break symmetry. Here we demonstrate high-multipole nonlinear optical polarimetry as a sensitive and mode selective probe of SRO induced distortions using CrSiTe_3_ as a testbed. This compound is composed of weakly bonded sheets of nearly isotropic ferromagnetically interacting spins that, in the Heisenberg limit, would individually be impeded from LRO by the Mermin-Wagner theorem. Our results show that CrSiTe_3_ evades this law via a two-step crossover from two- to three-dimensional magnetic SRO, manifested through two successive and previously undetected totally symmetric distortions above its Curie temperature.

## Abstract (web summary)

Exploring lattice distortions from magnetic short-range ordering (SRO) facilitates the understanding of magnetic long-range ordering (LRO). Here the authors apply high-multipole nonlinear optical polarimetry to track SRO induced distortions in CrSiTe_3_, showing that LRO is established via a crossover from two- to three-dimensional SRO.

## Introduction

**Fig. 1:**
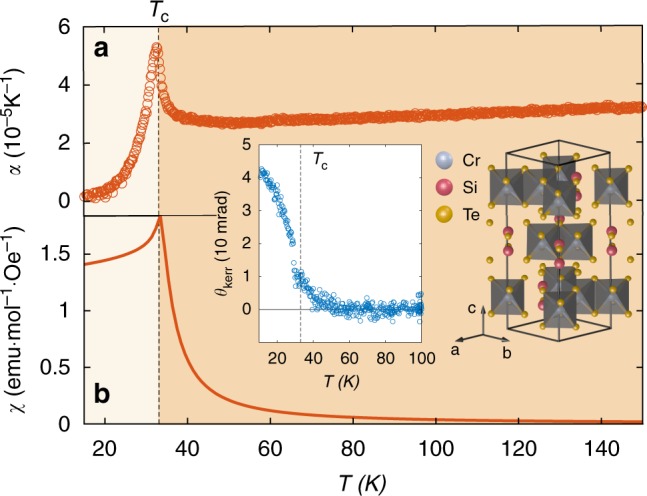
Characterization of long-range spin ordering in CrSiTe_3_. Temperature dependence of the **a** *c*-axis thermal expansion coefficient, **b** magnetic susceptibility and (left inset) magneto-optical Kerr rotation angle from a bulk single crystal of CrSiTe_3_ showing the clear onset of long-range magnetic order at *T*_c_ ~ 31 K. Note the absence of any features above *T*_c_ in all of these measurements. Right inset shows the crystal structure of CrSiTe_3_. In the FM phase, the Cr^3+^ spins all point parallel to the *c*-axis

Ferromagnetic (FM) semiconductors belonging to the transition metal trichalcogenide family have recently been shown to be promising starting materials for realizing monolayer ferromagnets by exfoliation^1,2^. However predicting the viability of the ferromagnetic long-range ordered state in the 2D limit relies on first understanding how FM long-range ordering (LRO) is established in the 3D bulk crystals, which is often unclear. A case in point is CrSiTe_3_^3–8^, which consists of *ABC* stacked sheets of Cr^3+^ (spin-3/2) moments arranged in a honeycomb network (Fig. 1). Each Cr atom is coordinated by six Te atoms that form an almost perfect octahedron^7^, giving rise to a near isotropic (Heisenberg) spin state and dominant FM nearest neighbor exchange interactions (*J*_ab  _ < 0) owing to the near 90° Cr–Te–Cr bond angle. This is corroborated by inelastic neutron scattering experiments^7^ on bulk CrSiTe_3_, which report a relatively feeble easy-axis (Ising) anisotropy strength (*D*/*J*_ab_ < 2 %). According to the Mermin-Wagner theorem^9^, LRO should be forbidden in a strictly 2D Heisenberg system. Therefore the finite value of the Curie temperature (*T*_c_ ~ 31 K) in bulk CrSiTe_3_ (Fig. 1) must either be driven by the weak spin anisotropy or by a weak interlayer coupling that mediates a crossover from 2D to 3D character. A dimensional crossover can in principle be uncovered by tracking the spatial anisotropy of short-range spin correlations using magnetic neutron and X-ray scattering techniques. However the requirement of nearly ideal bulk crystals and the difficulty of detecting and integrating diffuse magnetic scattering is restrictive and currently renders these techniques inoperable on exfoliated nanoscale thick sheets. Hence this mechanism is yet to be verified in CrSiTe_3_ or related compounds^1^.

An alternative route to measuring short-range spin correlations is through their effects on the crystal lattice. The magnetic energy of an insulating system is given by the thermal expectation value of its magnetic Hamiltonian ${\cal{H}}_m = J_{ij}\mathop {\sum }\nolimits_{i,j} \vec S_i \cdot \vec S_j$ which contains the short-range spin correlator $\langle\vec S_i \cdot \vec S_j\rangle$ as well as the exchange interaction *J*_*ij*_ between spins at sites *i* and *j*. Upon onset of magnetic short-range ordering (SRO), it may be energetically favorable for the system to re-adjust the distances and bonding angles between atoms that mediate *J*_*ij*_ in order to lower its magnetic energy, at the expense of some gain in elastic energy. Measuring such magneto-elastic distortions therefore yields information about spin correlations along various directions and, for simple low dimensional Hamiltonians, can even provide quantitative values of the $\langle\vec S_i \cdot \vec S_j\rangle$ function^10^, which is difficult to obtain by neutron scattering because only a limited range of its spatio-temporal Fourier components are accessed. However, SRO induced distortions are extremely hard to resolve because they are minute by virtue of $\langle\vec S_i \cdot \vec S_j\rangle$ being small, and because they generally do not break any lattice symmetries. A suitable probe must therefore be sensitive to and able to distinguish between different totally symmetric distortions (i.e., different basis functions of the totally symmetric irreducible representation). This suggests that examining the nonlinear high rank tensor responses of a crystal may be a promising approach.

Optical second harmonic generation (SHG), a frequency doubling of light produced by its nonlinear interactions with a material, is governed by high rank (>2) susceptibility tensors that are sensitive to many degrees of freedom in a crystal. Traditionally SHG has been exploited as a symmetry sensitive probe because the leading order electric dipole susceptibility necessarily vanishes if the system possesses a center of inversion. This makes SHG particularly powerful for studying surfaces of centrosymmetric crystals^11^, and for identifying bulk symmetry breaking phase transitions through the appearance of additional, high symmetry forbidden, tensor elements^12–15^. In principle, SHG can also be utilized to study symmetry preserving distortions^16^ by examining their subtle effects on the existing symmetry allowed tensor elements. However this potential capability is highly underexplored, in part due to the technical demand of simultaneously tracking small changes across an entire set of allowed tensor elements.

**Fig. 2:**
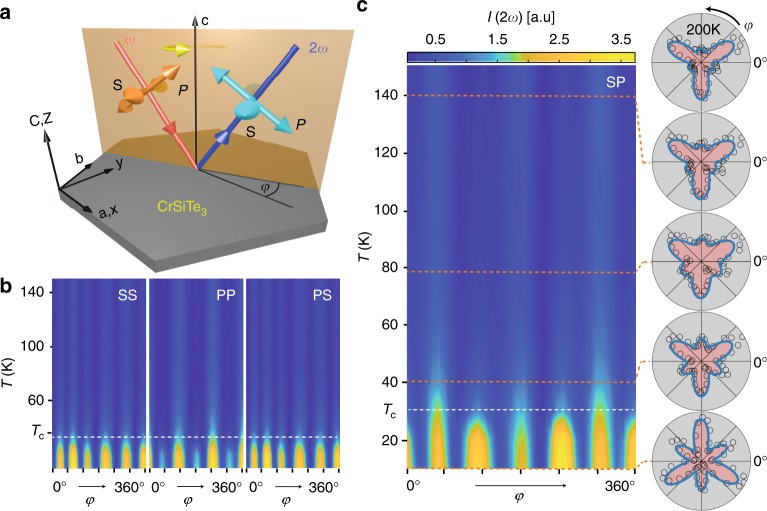
Temperature dependence of the SHG rotational anisotropy. **a** Experimental geometry of the RA SHG experiment. The *φ* = 0° direction coincides with the crystallographic *a*-axis. **b** Image plots of the raw temperature dependent RA SHG data acquired in SS, PP, and PS polarization configurations, where the first and second labels denote the selected incident (ω) and detected (2ω) polarization components. The horizontal black line indicates the *T*_c_ of our sample (see Fig. 1). **c** A zoom-in on the RA SHG image plot acquired in SP configuration. Cuts at select temperatures displayed as polar plots (open circles) are shown to emphasize the changes in shape of the RA patterns taking place above *T*_c_. Blue curves are best fits to the expected EQ SHG response from a $\bar 3$ point group as described in the text

Recently we managed to surmount this challenge by developing a rotating scattering plane based SHG polarimetry technique^17^. In these experiments, linear (either P or S) polarized light of frequency ω is focused obliquely onto the surface of a bulk single crystal. The intensity of either the P or S component of reflected light at frequency 2ω is then measured as a function of the angle (φ) that the scattering plane is rotated about the *c*-axis (Fig. 2a), which allows a multitude of SHG susceptibility tensor elements to be sampled. By collecting these rotational anisotropy (RA) patterns with different polarization combinations, a complete set of SHG susceptibility tensor elements can typically be uniquely determined. Here we apply this technique to track the magnitudes of all of the symmetry allowed SHG susceptibility tensor elements of CrSiTe_3_ as a function of temperature. Evidence of previously undetected structural distortions are observed above *T*_c_ at *T*_2D_ ~ 110 K and *T*_3D_ ~ 60 K. Using a hyper-polarizable bond model, we are able to attribute the distortions at *T*_2D_ and *T*_3D_ to displacements along different totally symmetric normal mode coordinates, which are consistent with an onset of intralayer and interlayer spin correlations respectively.

## Results

### SHG polarimetry results

The full temperature evolution of the RA patterns acquired from the (001) surface of CrSiTe_3_ under select polarization geometries is displayed in Fig. 2b. We first note that a finite weak SHG intensity is present at all temperatures despite previous work showing that CrSiTe_3_ always retains a centrosymmetric structure with $\bar 3$ point group symmetry^18^. This suggests that the SHG originates from a higher multipole process such as electric quadrupole (EQ) radiation, which is governed by a fourth rank susceptibility tensor $\chi _{ijkl}$ that has only eight independent non-zero elements ($\chi _{xxxz}$, $\chi _{xxyy}$, $\chi _{xxzz}$, $\chi _{yxxx}$, $\chi _{yyyz}$, $\chi _{zzxx}$, $\chi _{zzxy}$, $\chi _{zzzz}$) after accounting for the symmetries of the $\bar 3$ point group and the degeneracy of the incident electric fields^19^. Expressions for the RA SHG intensity $I\left( {2\omega } \right) \propto \left| {\hat e_i^{2\omega }\left( \varphi \right)\chi _{ijkl}\hat e_j^\omega \left( \varphi \right)\kappa _k\left( \varphi \right)\hat e_l^\omega \left( \varphi \right)} \right|^2I_0^2\left( \omega \right)$ derived under these conditions (here $\hat e$ are the polarization directions, $\kappa$ is the incident wave vector and $I_0$ is the incident intensity; see Supplementary Note 1) indeed produce excellent fits to our set of RA patterns at any given temperature and allow us to uniquely determine the values of $\chi _{ijkl}$ at each temperature. In contrast, other possible allowed SHG processes such as surface electric dipole or bulk magnetic dipole radiation cannot reproduce the RA data and are thus treated as negligibly small (see Supplementary Note 2).

From the raw RA data (Fig. 2b) we can clearly discern the bulk three-fold rotational symmetry of CrSiTe_3_ and, as expected, we observe no change in symmetry as a function of temperature. Yet the absolute and relative intensities of the various features do undergo changes upon cooling, which must encode symmetry preserving distortions. Most notably, there is a dramatic increase of intensity below *T*_c_ that, as we will show later on, arises from LRO induced magneto-elastic distortions that have previously been detected by optical absorption^20^, Raman scattering^20^, and X-ray diffraction^18^, and are also captured by our dilatometry measurements (Fig. 1a). Surprisingly however, we find that the RA patterns continue to subtly evolve even far above *T*_c_. In SP polarization geometry for example (Fig. 2c), representative RA patterns at 140, 80, and 40 K have qualitatively different shapes, indicating that the magnitude of the $\chi _{ijkl}$ elements change non-uniformly with temperature.

### Temperature dependence of the nonlinear susceptibility

**Fig. 3:**
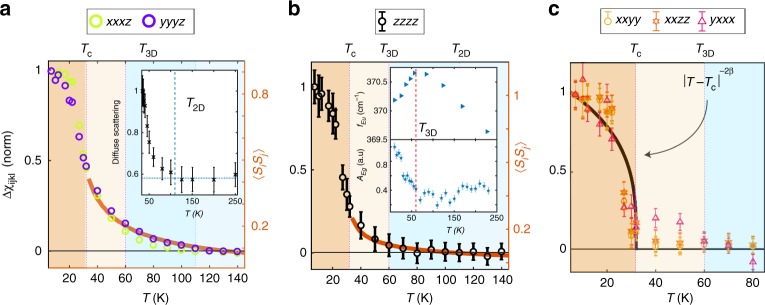
Temperature evolution of individual susceptibility tensor elements. The values of the **a** *xxxz*, *yyyz*, **b** *zzzz* and **c** remaining elements of the SHG susceptibility tensor $\chi _{ijkl}$ at each temperature extracted through simultaneous fitting to all four polarization configurations of the RA data (see text). For every element, a high temperature background derived from the data above *T* = 150 K was subtracted off. The background subtracted curves ($\Delta \chi _{ijkl}$) were then each normalized to their values at *T* = 7 K. Error bars are the least squared errors of the fits, which are smaller than the data symbols in panel **a**. Solid lines in panels **a** and **b** are the calculated intralayer and interlayer spin correlator respectively. The line in panel **c** is the function $\left| {T - T_c} \right|^{ - 2\beta }$, where *β* is the critical exponent of the magnetization. The two elements *zzxx* and *zzxy* are not displayed in **c** for clarity because their fitted values are very small, resulting in much larger error bars. However their trend follows that of the *xxyy*, *xxzz* and *yxxx* elements shown in **c** (see Supplementary Note 4). Diffuse magnetic neutron scattering data from CrSiTe_3_ reproduced from ref. ^7^ is shown in the inset of **a**. The inset of **b** shows the frequency of the *E*_u_ phonon (top) and amplitude of the *E*_g_ phonon (bottom), which are respectively reproduced from ref. ^20^ and measured using impulsive stimulated Raman scattering (see Supplementary Note 7)

The temperature (*T*) dependence of each of the eight individual $\chi _{ijkl}$ elements was extracted through the aforementioned fitting procedure (Fig. 2c). From every $\chi _{ijkl}\left( T \right)$ curve, we subtracted a high temperature background using the data above 150 K, where the shapes of the RA patterns have ceased evolving (see Supplementary Note 3). Figure 3 shows the complete set of background subtracted curves ${\mathrm{\Delta }}\chi _{ijkl}\left( T \right)$ that have all been normalized to their low temperature values. Three distinct sets of behavior are clearly resolved. Below a characteristic temperature *T*_2D_ ~ 110 K, the *xxxz* and *yyyz* elements alone start to grow in tandem (Fig. 3a). Then below a second characteristic temperature *T*_3D_ ~ 60 K, a solitary *zzzz* element begins to grow (Fig. 3b). The temperature dependence of the former and latter set of elements are sub-linear above *T*_c_ and scale with classical calculations of the nearest neighbor intralayer and interlayer spin correlators respectively (see Supplementary Note 5). By contrast, below *T*_c_ the remaining five elements turn up with an order parameter like temperature dependence indicative of a phase transition (Fig. 3c), with a critical exponent twice that reported^7^ for the magnetization (2*β* ≈ 0.3). Since magneto-elastic distortions scale like the square of the magnetic order parameter, this further confirms that $\chi _{ijkl}$ is probing the lattice degrees of freedom. This also shows that $\chi _{ijkl}$ is a time-reversal invariant *i*-tensor^19^, which naturally explains why our measurements are insensitive to magnetic domains^21^.

### Microscopic origin of the susceptibility change

To understand the microscopic origin of the features in ${\mathrm{\Delta }}\chi _{ijkl}\left( T \right)$, we appeal to a simplified hyper-polarizable bond model^22^, which treats the crystal as an array of charged anharmonic oscillators centered at the chemical bonds and constrained to only move along the bond directions. The nonlinear polarizability of each oscillator is calculated by solving classical equations of motion, and then appropriately summed together to form the total nonlinear susceptibility. Recently an expression for the EQ SHG susceptibility was derived using this model^23^ and was found to take the form $\chi _{ijkl} \propto \mathop {\sum }\nolimits_n \alpha _\omega \alpha _{2\omega }\left( {\hat b_n \otimes \hat b_n \otimes \hat b_n \otimes \hat b_n} \right)_{ijkl}$, where $\alpha _\omega$ and $\alpha _{2\omega }$ are the first-order (linear) and second-order (hyper) polarizabilities, $\hat b_n$ is a unit vector that points along the *n*^th^ bond, and all bond charges are assumed equal. Using this expression, we investigated how distortions along each of the four totally symmetric normal mode coordinates allowed in the $\bar 3$ point group (i.e., the four basis functions $A_g^1$, $A_g^2$, $A_g^3$ and $A_g^4$ of its totally symmetric irreducible representations) change the individual $\chi _{ijkl}$ elements.

**Fig. 4:**
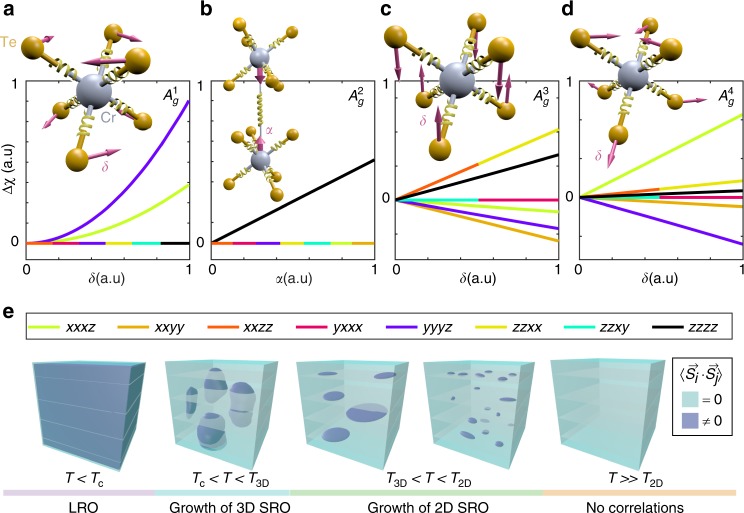
Hyper-polarizable bond model and dimensional crossover. Plots show hyper-polarizable bond model calculations of the change in each of the eight allowed SHG susceptibility tensor elements relative to their undistorted values (${\mathrm{\Delta }}\chi _{ijkl}$) under totally symmetric distortions along the **a**, $A_g^1$, **b**, $A_g^2$, **c**, $A_g^3$ and **d**, $A_g^4$ normal coordinates. Schematics in the insets show the bonds (springs) used in our model and depict how each distortion is parameterized. Both vertical and horizontal axes are plotted on a linear scale. Tensor elements that change in the same way are represented as multi-colored curves. **e** Illustration of the spin ordering process in CrSiTe_3_ deduced from our data. The light blue regions represent an absence of spin-spin correlations and the dark blue regions represent FM correlations, which are dynamic above *T*_c_ and static below *T*_c_

For simplicity, we considered only the nearest neighbor intralayer Cr–Te bonds and the nearest neighbor interlayer Cr–Cr bonds, which is reasonable because the states accessed by our photon energy (2ħω = 3 eV) are predominantly composed of Cr and Te orbitals^24,25^. Remarkably, our hyper-polarizable bond model shows that under a small distortion along the $A_g^1$ normal coordinate δ, which we implement by changing the $\hat b_n$ while keeping the $\alpha _\omega \alpha _{2\omega }$ values constant, only the *xxxz* and *yyyz* elements are affected (Fig. 4a), in perfect agreement with our observations below *T*_2D_ (Fig. 3a). Since motion along $A_g^1$ deforms the Te octahedra and can bring the Cr–Te–Cr bond angle closer to 90° to strengthen *J*_ab_, it is natural to associate this distortion with the development of FM in-plane spin correlations. This is further supported by neutron scattering experiments^7^, which show a rise in magnetic diffuse scattering around *T*_2D_ (inset Fig. 3a) indicative of a growing in-plane correlation length $\xi _{ab}$.

To uncover a mechanism that would exclusively affect the *zzzz* element below *T*_3D_ (Fig. 3b), we note that the distortion along the $A_g^2$ normal coordinate involves a pure out-of-plane displacement of the Cr atoms. Although this motion does not change the $\hat b_n$ of the interlayer Cr–Cr bonds since they remain parallel to the *z*-axis, it will change their polarizabilities by virtue of their altered bond length. Assuming that it is these Cr–Cr bonds that primarily contribute to the observed changes at *T*_3D_ (see Supplementary Note 6), our model indeed shows that tuning either $\alpha _\omega$ or $\alpha _{2\omega }$ of the Cr–Cr bond will exclusively affect the *zzzz* element (Fig. 4b). This naturally suggests an association of the $A_g^2$ distortion with the development and enhancement of FM interlayer spin correlations, and hence an identification of *T*_3D_ as the 2D to 3D dimensional crossover temperature. Independent evidence for a structural distortion at *T*_3D_ was also found via anomalies in the *E*_g_ and *E*_u_ phonons using impulsive stimulated Raman scattering (see Supplementary Note 7) and infrared absorption measurements^20^ respectively (inset Fig. 3b), which likely arise from their nonlinear coupling to the $A_g^2$ distortion.

As a further consistency check, we note that one expects interlayer correlations to onset when $\xi _{ab}$ grows to a size where the total interlayer exchange energy becomes comparable to the temperature. In a mean field approximation, this condition is expressed as $T = N\left( T \right)J_cS\left( {S + 1} \right)/3k_B$, where *N* is the number of in-plane correlated spins of magnitude *S* that are interacting with the next layer, *J*_c_ is the interlayer Cr–Cr exchange and $k_B$ is Boltzmann’s constant. Using the values of $\xi _{ab}\left( T \right)$ and *J*_c_ determined from neutron scattering^7^, we find a solution to the mean field equation at *T* ~ 70 K (see Supplementary Note 8), which is reasonably close to *T*_3D_. Displacements along the remaining two $A_g^3$ and $A_g^4$ normal coordinates are found from our model to affect all eight of the tensor elements (Fig. 4c, d) and are therefore not measurably induced at either *T*_2D_ or *T*_3D_. It is possible that they occur below *T*_c_ where we observe all elements to change (Fig. 3), but details of LRO induced distortions are outside the scope of this work.

## Discussion

Our EQ SHG data and analysis taken together provide a comprehensive picture of how the quasi-2D Heisenberg ferromagnet CrSiTe_3_ evades the Mermin-Wagner theorem via a multiple stage process to establish long-range spin order (Fig. 4e), and shows that interlayer interactions are vital to stabilizing LRO at such high temperatures. More generally, our results demonstrate that the nonlinear optical response is a highly effective probe of short-range spin physics and their associated totally symmetric magneto-elastic distortions, which are typically unresolvable by capacitance dilatometry^10^ (Fig. 1a) or lower rank optical processes like linear reflectivity and Raman scattering due to their limited degrees of freedom (see Supplementary Note 9), and are challenging to detect by diffraction based techniques limited to pico-meter resolution^18^. This technique will be particularly useful for studying anisotropic or geometrically frustrated magnetic systems, which tend to display interesting short-range spin correlations. It will also be useful for uncovering magnetic ordering mechanisms in monolayer or few layer ferromagnetic and antiferromagnetic nanoscale flakes and devices^26–29^, which are often unclear because of their inaccessibility by neutron diffraction. We anticipate that access to this type of information may offer new strategies to control magnetism based on manipulating SRO induced distortions through chemical synthesis, static perturbations or even out-of-equilibrium excitations^30^.

## Methods

### Sample growth and characterization

The CrSiTe_3_ crystals used in this study were grown using a Te self-flux technique^20^. High purity Cr (Alfa Aesar, 99.999%), Si (Alfa Aesar 99.999%), and Te (Alfa Aesar 99.999%) were weighed in a molar ratio of 1:2:6 (Cr:Si:Te) and loaded into an alumina crucible sealed inside a quartz tube. The quartz ampoule was evacuated and backfilled with argon before sealing. Plate-like crystals up to 5 mm thick with flat, highly reflective surfaces were then removed from the reaction crucible. X-ray diffraction (XRD) data collected on crushed crystals using an Emperyan diffractometer (Panalytical) confirmed the correct $R\bar 3$, space group 148, CrSiTe_3_ phase. Measurements of the temperature and field-dependence of the magnetization were carried out using a Magnetic Property Measurement System (MPMS, Quantum Design). The samples were mounted with the field applied parallel to the *ab*-plane of the crystals for magnetization and susceptibility measurements. The thermal expansion coefficient was measured using the Quantum Design dilatometer option in a PPMS DynaCool. Dilation was measured along the *c*-axis; sample thickness in this direction was 0.41 mm. Data were collected under a ramp rate of 0.1 K/min.

### RA SHG measurements

Incident light with <100 fs pulse width and 800 nm center wavelength was derived from a ti:sapph amplified laser system (Coherent RegA) operating at 100 kHz. Specular reflected second-harmonic light at 400 nm was selected using short-pass and narrow bandpass filters and measured with a two-dimensional EM-CCD camera (Andor iXon Ultra 897). Both the sample and detector remained fixed while the scattering plane is rapidly mechanically spun about the central beam axis. The angle of incidence was fixed at 10°. A detailed description of the RA SHG apparatus used can be found in ref. ^17^. The fluence of the beam was maintained at ~340 μJ cm^−2^ with a spot size of ~30 μm FWHM. The close agreement between the *T*_c_ values measured using RA SHG and magnetic susceptibility indicates negligible average heating by the laser beam. Each complete RA pattern was acquired with a 5 min exposure time. Samples (~1 mm × 2 mm × 0.1 mm) were cleaved prior to measurement and immediately pumped down in an optical cryostat to a pressure better than 10^−6^ Torr.

## Supplementary information

Supplementary Information

Peer Review

## Data Availability

The datasets generated are/or analyzed during the current study are available from the corresponding author on reasonable request.
