# Supplementary material for: Dimensional crossover in a layered ferromagnet detected by spin correlation driven distortions
Source: Nat Commun. 2019 Apr 10;10:1654. doi: 10.1038/s41467-019-09663-3 (PMC6458139; doi:10.1038/s41467-019-09663-3)
Supplement: Supplementary file 1 — Supplementary Information [file 41467_2019_9663_MOESM1_ESM.pdf]

Supplementary Information for

Dimensional crossover in a layered ferromagnet detected by spin correlation driven distortions

A. Ron et al.

Contents:

Supplementary Note 1. Mathematical expressions for EQ induced RA-SHG patterns
Supplementary Note 2. Best fits to alternative SHG processes
Supplementary Note 3. High temperature background subtraction procedure and fits
Supplementary Note 4. Temperature dependence of χ_{zzxx} and χ_{zzxy}
Supplementary Note 5. Classical Heisenberg model calculations
Supplementary Note 6. Effect of A_{g}^2 distortion on Cr-Te bond contribution to $\Delta\chi_{ijkl}$
Supplementary Note 7. Details of impulsive stimulated Raman scattering measurements
Supplementary Note 8. Mean-field estimate of T_{3D}
Supplementary Note 9. Bond model results for a rank-2 tensor response

Supplementary Note 1. Mathematical expressions for EQ induced RA-SHG patterns

Expressions for the bulk EQ derived RA-SHG intensity I from a crystal with $\bar{3}$ point group symmetry for the four experimental polarization configurations are given below, where θ = 10° is the experimental angle of incidence. The RA-SHG data were simultaneously fit to all four expressions to extract the values of the susceptibility tensor elements.

$$I_{PP} = \cos^2\theta\Big(\Big(\sin^2\theta\chi_{zzxx}\cos^2\theta - \chi_{xxxz}\cos\theta\sin\theta\cos(3\varphi) - 2\chi_{zzxx}\sin^2\theta + \chi_{zzzz}\sin^2\theta - \\ -\frac{1}{2}\chi_{yyyz}\sin(2\theta)\sin(3\varphi)\Big)^2 + \Big(\chi_{xxxz}\cos\theta\cos(3\varphi)\big(\cos^2\theta - 2\sin^2\theta\big) - \\ -\sin\theta\big(3\chi_{xxyy}\cos^2\theta - 2\chi_{xxzz}\cos^2\theta + \chi_{xxzz}\sin^2\theta\big) + \\ +\chi_{yyyz}\big(\cos^3\theta - \sin\theta\sin(2\theta)\big)\sin(3\varphi)\Big)^2\Big) \tag{1}$$

$$I_{PS} = \Big(\chi_{yyyz}\cos\theta\cos(3\varphi)\big(\cos\theta - 2\sin^2\theta\big) + \sin\theta\big(\chi_{yxxx}\cos^2\theta + 2\chi_{zzxy}\cos^2\theta - \chi_{zzxy}\sin^2\theta\big) + \\ +\chi_{xxxz}\big(-\cos^3\theta + \sin\theta\sin(2\theta)\big)\sin(3\varphi)\Big)^2 \tag{2}$$

$$I_{SP} = \cos^2\theta\big(\chi_{xxxz}\cos\theta\cos(2\varphi) + \chi_{xxyy}\sin\theta + \chi_{yyyz}\cos\theta\sin(3\varphi)\big)^2 + \\ +\sin^2\theta\big(\chi_{zzxx}\cos\theta + \chi_{xxxz}\cos(3\varphi)\sin\theta + \chi_{yyyz}\sin\theta\sin(3\varphi)\big)^2 \tag{3}$$

$$I_{SS} = \big(\chi_{yxxx}\sin\theta + \cos\theta\big(\chi_{yyyz}\cos(3\varphi) - \chi_{xxxz}\sin(3\varphi)\big)\big)^2 \tag{4}$$

Supplementary Note 2. Best fits to alternative SHG processes

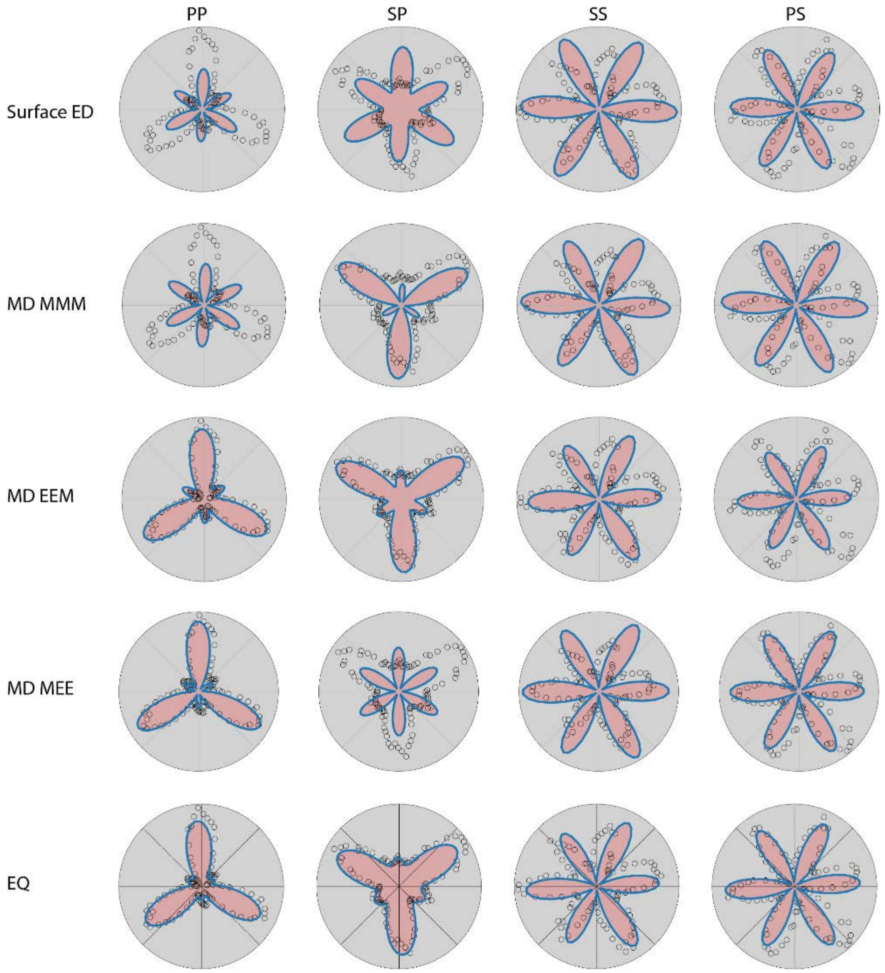

Supplementary Figure 1. Best fits of the RA-SHG data (circles) at *T* = 200 K to the following SHG processes (blue curves): (from top to bottom) surface ED, bulk MD (MMM), bulk MD (EEM), bulk MD (MEE) and bulk EQ. Simultaneous fits were made to the data in four different polarization configurations: PP, SP, SS and PS. The intensity in the SS and PS channels is ~ 50 % smaller than in the PP and SP channels, which is the reason for the larger scatter in those data sets.

We examined all of the leading order contributions to SHG allowed in a centrosymmetric crystal: the surface electric-dipole (ED) process given by $P_i = \chi_{ijk}^{EEE} E_j E_k$, three bulk magnetic-dipole (MD) processes given by $M_i = \chi_{ijk}^{MMM} H_j H_k$, $P_i = \chi_{ijk}^{EEM} E_j H_k$ and $M_i = \chi_{ijk}^{MEE} E_j E_k$, and a bulk electric-quadrupole (EQ) process given by $P_i = \chi_{ijkl}^{QEE} E_j \nabla_k E_l$, where *P* and *M* are the induced polarization and magnetization, and *E* and *H* are the incident electric and magnetic fields respectively. Representative best fits of each of these processes to our RA-SHG data are shown in Supplementary Figure 1. Clear discrepancies are observed for the surface ED and bulk MMM and MEE processes and are therefore immediately ruled out. Discrepancies in the bulk EEM fits

also exist but are more subtle. Most notably, the small lobes in both PP and SP polarizations are not adequately captured over most of the temperature range of the experiment. Although these discrepancies in the bulk EEM fits are difficult to notice in the polar plots shown in Supplementary Figure 1, they are clear when plotted in Cartesian form (Supplementary Figure 2).

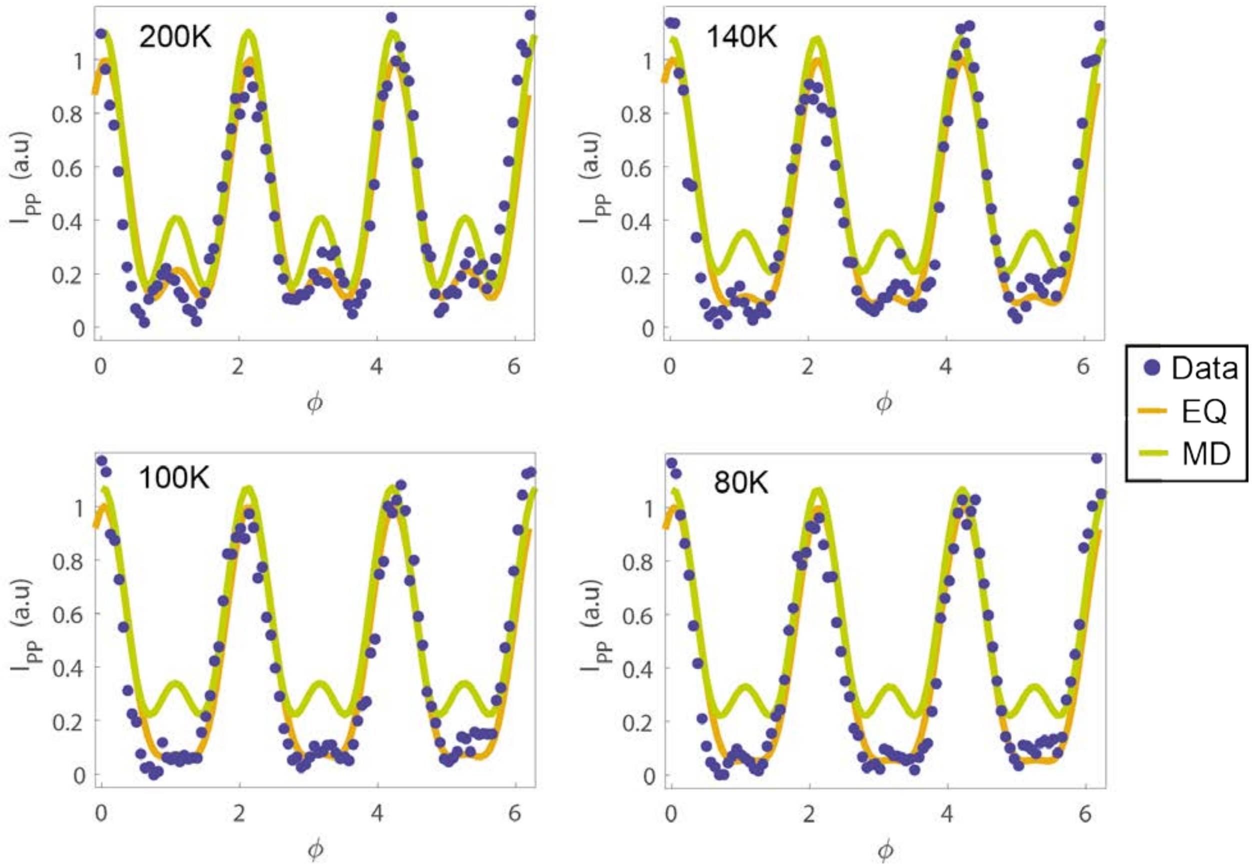

Supplementary Figure 2. Best fits to the RA-SHG data (blue circles) for the EQ (orange line) and MD EEM (green line) processes at various temperatures for the PP configuration. It is clear that the EQ process provides good fits to the data whereas the MD process does not.

Supplementary Note 3. High temperature background subtraction procedure and fits

To obtain the curves shown in Figure 3 of the main text, we first extracted the temperature dependence of each susceptibility tensor element by fitting the expressions given in Supplementary Note 1 to the raw RA-SHG data. For each tensor element independently, we then subtracted a weakly linear background using the data above 150 K where the shapes of the RA-SHG patterns have ceased changing (i.e. far above where magnetic correlations start to affect our data). In Supplementary Figure 3 we explicitly show that over the temperature range used for estimating the background, there is indeed no measureable change in shape of the RA-SHG patterns. The direct fit results before any background subtraction or normalization is performed are shown in Supplementary Figure 4.

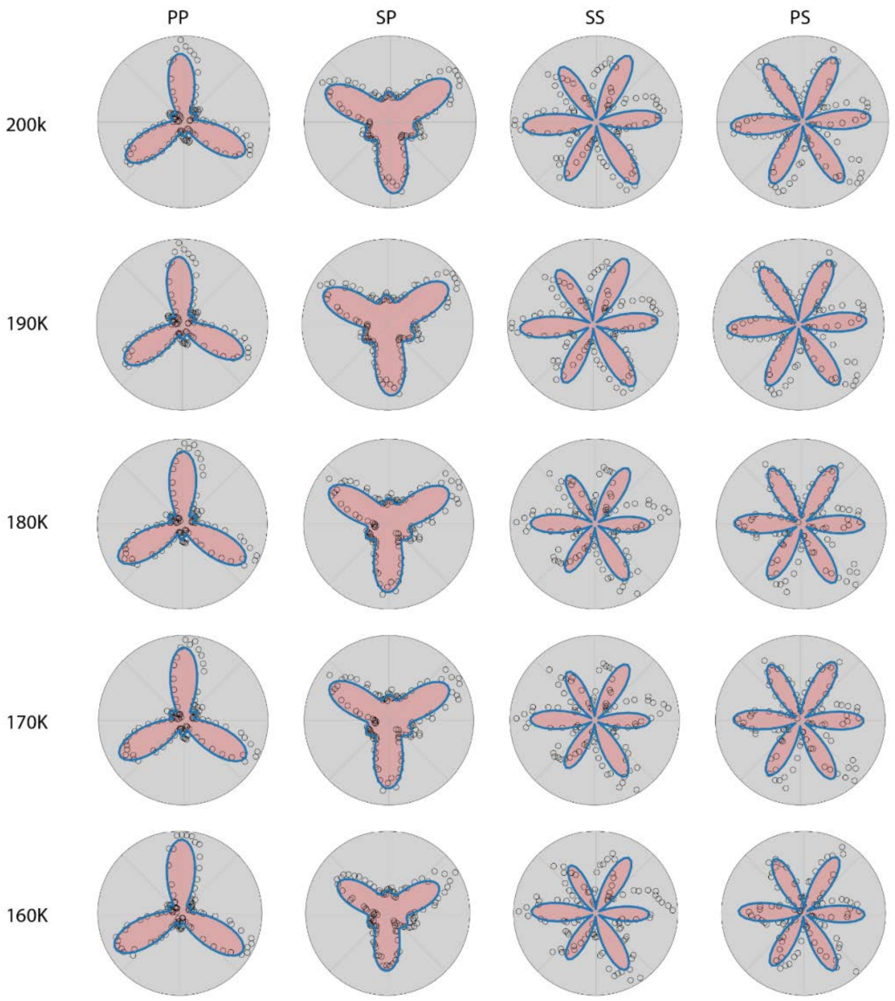

Supplementary Figure 3. RA-SHG data (circles) and EQ fits (blue curves) at various temperatures above 150 K.

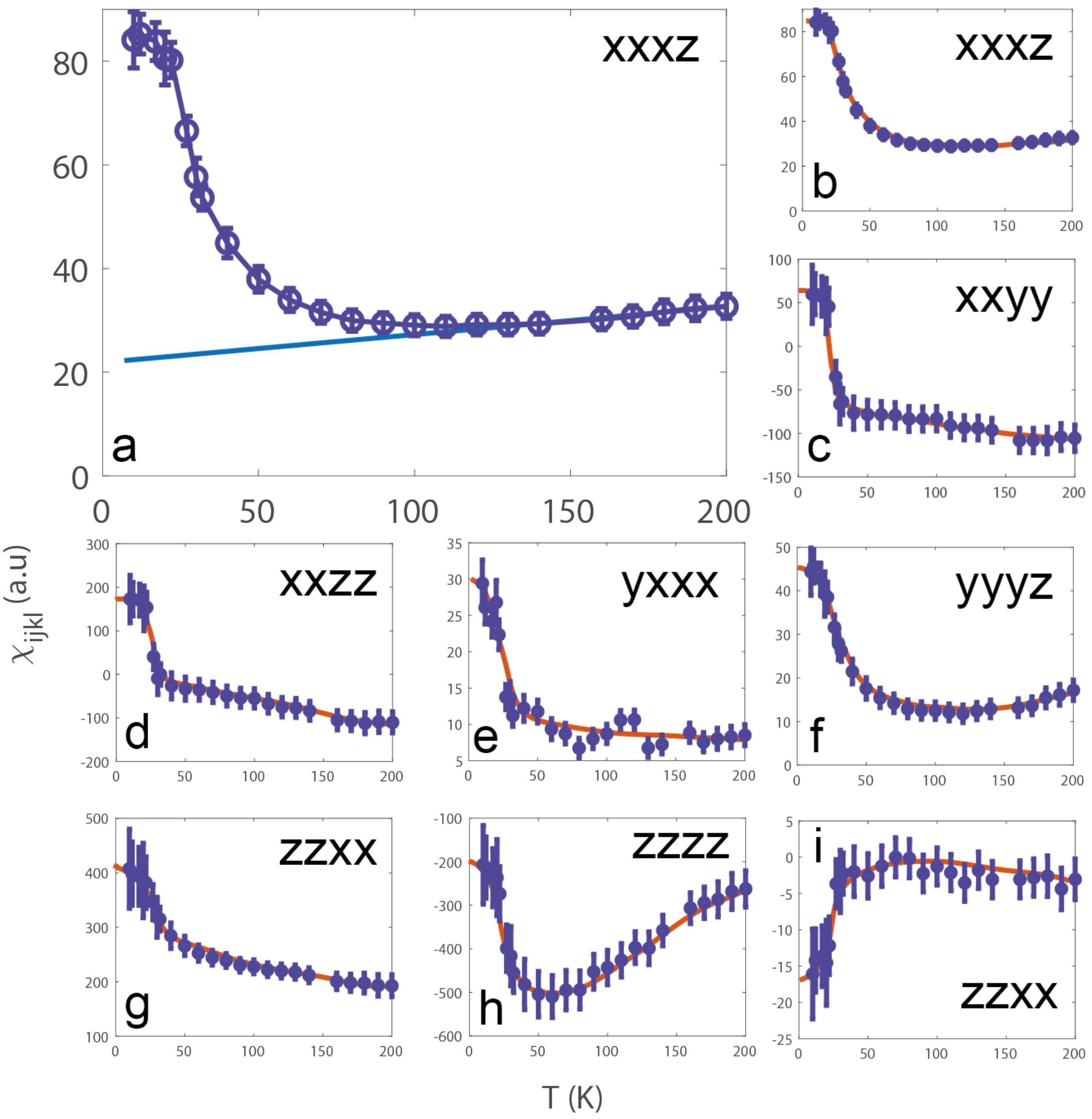

Supplementary Figure 4. Temperature dependence of the various tensor elements obtained directly from fitting the RA-SHG data. Error bars are the least squared errors of the fits. The blue curve in **a** is a linear fit to the data above 150 K, which is then subtracted off to produce the $\Delta\chi_{ijkl}$ curves shown in Fig. 3 of the main text. The same procedure was independently performed for all 8 tensor elements. The results of the fitting procedure without any background subtraction or normalization are shown in panels **b-i** for all tensor elements. Red lines are guides to the eye.

Supplementary Note 4. Temperature dependence of χ_{zzxx} and χ_{zzxy}

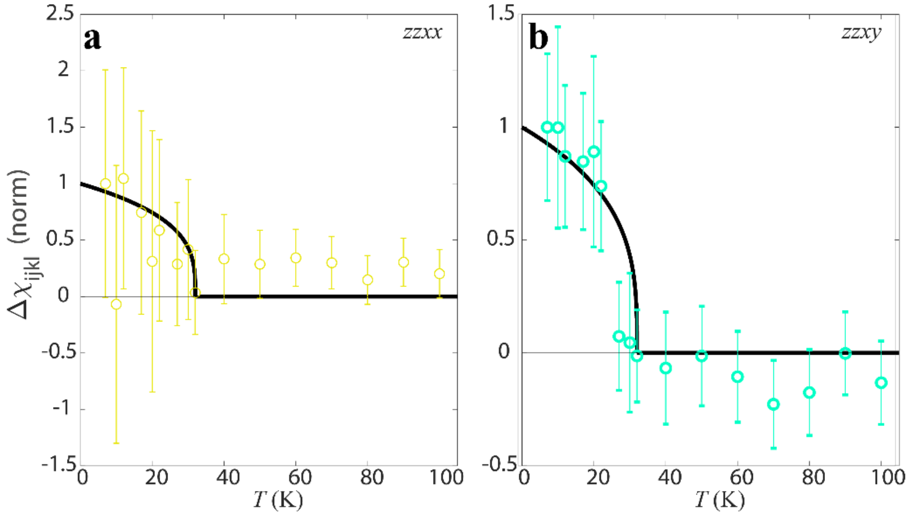

Supplementary Figure 5. Normalized and background subtracted temperature dependence of the **a,** *zzxx* and **b,** *zzxy* tensor elements, which are not shown in Fig. 3c of the main text because their absolute values are small, leading to much larger error bars than the other tensor elements. These two elements behave like the *xxyy*, *xxzz* and *yxxx* elements, turning up only below T_c. The black lines are proportional to the function $|T - T_c|^{-2\beta}$ where β is the critical exponent of the magnetization.

Supplementary Note 5. Classical Heisenberg model calculations

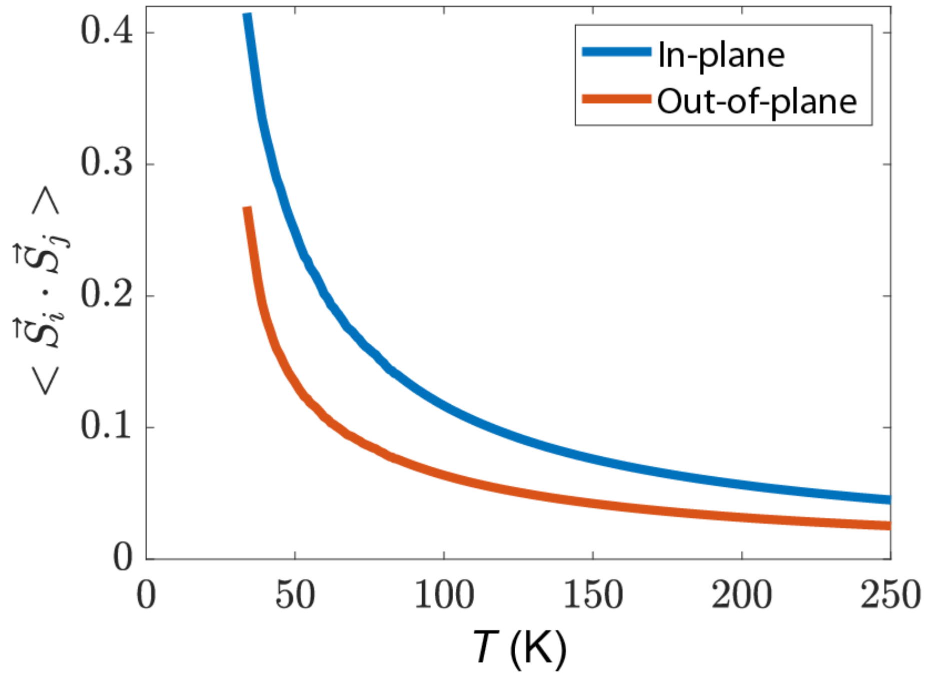

Supplementary Figure 6. Temperature dependence of the nearest neighbor intralayer (blue) and interlayer (orange) spin correlators calculated by solving the classical Heisenberg model in a large-N approximation using the experimental parameters for $CrSiTe_3$.

To understand the temperature dependence of the short-range spin correlations in $CrSiTe_3$, we calculated the nearest neighbor correlations from a classical Heisenberg model, treating the spins as vectors of length $S = 3/2$ and using the exchange parameters from Ref. [7]. To calculate the correlations, we used a large-N approximation, expected to be reasonably accurate above the critical temperature. Specifically, the fixed length constraint is implemented on average via a Lagrange multiplier $\lambda(T)$, which is self-consistently determined at each temperature. The spin correlations were then obtained as the Fourier transform of the inverse exchange interaction matrix, shifted appropriately by $\lambda(T)$. This approach gives $T_c = 33.8$ K, which agrees excellently with experiment and serves as a further self-consistency check of the dimensional crossover picture. Supplementary Figure 6 shows the calculated temperature dependence of the nearest neighbor intralayer and nearest neighbor interlayer spin correlator, which shows a sub-linear temperature dependence that is consistent with that of the *xxxz*, *yyyz* and *zzzz* susceptibility tensor elements shown in Figure 3 of the main text.

Supplementary Note 6. Effect of A_g^2 distortion on Cr-Te bond contribution to $\Delta\chi_{ijkl}$

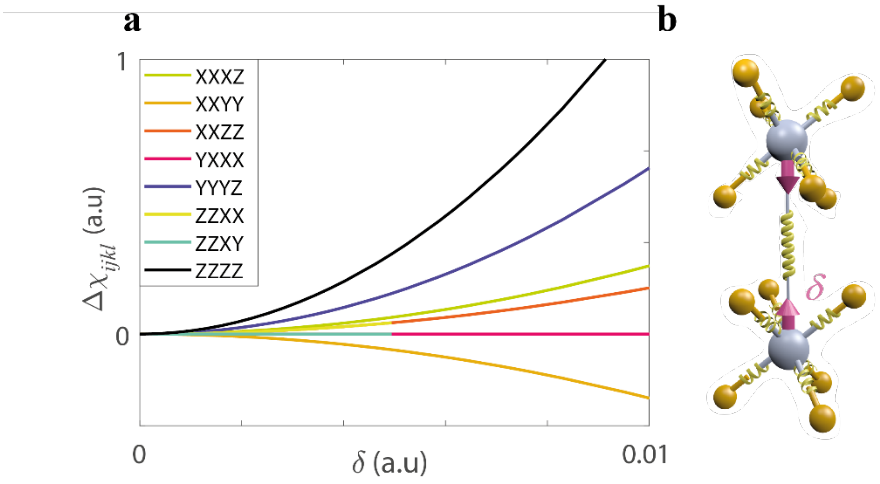

Supplementary Figure 7. **a,** Change in the susceptibility tensor elements due to a displacement along the A_g^2 normal coordinate calculated using the hyper-polarizable bond mode including only the Cr-Te bonds. **b,** Schematic of the displacement δ of the Cr atoms.

In Figure 4b of the main text, we showed how a displacement along the A_g^2 normal coordinate affects the interlayer Cr-Cr bond contribution to χ_{ijkl}. Here we show how this distortion affects the intralayer Cr-Te bond contribution to χ_{ijkl} through a modification of the $\hat{b}_n$ associated with the Cr-Te bonds. As shown in Supplementary Figure 7, it is again the *zzzz* element that is primarily affected, consistent with our experiments (Fig. 3b). However, in this case the *xxxz, yyyz, xxzz, zzxx* and *xxyy* elements are also affected to a smaller degree, which we cannot resolve experimentally. Therefore we conclude that the Cr-Cr bond contribution dominates over the Cr-Te contribution for the A_g^2 distortion.

Supplementary Note 7. Details of impulsive stimulated Raman scattering measurements

To independently verify the presence of a structural distortion at T_{3D}, we performed impulsive stimulated Raman scattering (ISRS) measurements on $CrSiTe_3$ to track the behavior of Raman active phonons as a function of temperature. In our experiment, the sample was excited using an ultrashort (< 100 fs) optical pump pulse at a wavelength of 1200 nm, which excites coherent phonons through the ISRS mechanism. Phonon vibrations were then resolved in the time-domain by measuring the instantaneous polarization rotation angle θ of a reflected optical probe pulse at a wavelength of 800 nm as a function of time-delay *t*. As shown in Supplementary Figure 8, traces of θ(*t*) exhibit clear oscillations due to coherent phonons and are dominated by two frequency components at ~3.7 THz and ~2.8 THz, which correspond to the frequencies of the A_g and E_g modes respectively. The amplitudes of the modes were determined by the peak intensities of the Fourier transform (inset Supplementary Figure 8) and tracked as function of temperature. A clear anomaly in the amplitude of the E_g mode was observed at T_{3D} (inset Fig. 3b). No clear anomaly at T_{3D} was found in the A_g mode, likely indicating a weaker nonlinear coupling to the A_g^2 distortion.

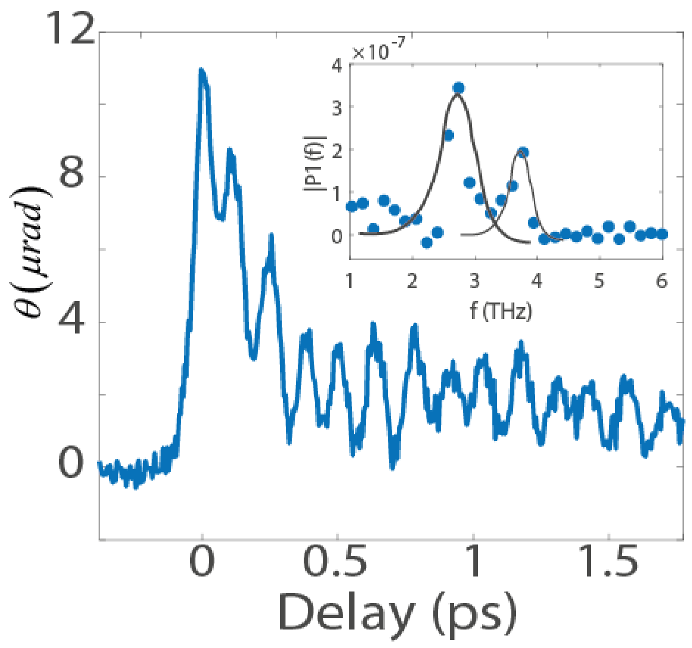

Supplementary Figure 8. Time-resolved polarization rotation of $CrSiTe_3$ acquired at T = 15 K showing a beat pattern coming from the E_g and A_g phonon modes. The two frequency components are more clearly resolved in the Fourier transform (inset).

Supplementary Note 8. Mean-field estimate of T_{3D}

We expect the interlayer correlations to onset when the total interlayer exchange energy becomes comparable to the temperature. In a mean field approximation this condition is expressed as $T = N(T)J_cS(S+1)/3k_B$. where N is the number of in-plane correlated spins of magnitude S that are interacting with the next layer, J_c is the interlayer Cr-Cr exchange and k_B is Boltzmann's constant. When counting $N(T)$, it is important to note that there are two inequivalent Cr sites due to the *ABC* type stacking (Supplementary Figure 9 inset). One type of site has a Cr ion directly above and a Si pair directly below, while the other type of site has the reversed situation. Each site occupies one of the two sub-lattices of the honeycomb structure. When calculating the total interlayer exchange energy one should therefore count only Cr ions on the same type of site (i.e. sub-lattice, see Supplementary Figure 9 inset) because only they couple to the same adjacent layer. The Cr ions that sit on a different sub-lattice are coupled through J_c to a different layer. Since it is only the second nearest neighbors (2NN) that sit on the same type of site, when the in-plane spin correlation length $\xi_{ab}(T) < d_{2NN}$, $N(T) = 1$ (only the Cr ion itself), and when $d_{2NN} < \xi_{ab}(T) < d_{3NN}$, $N(T) = 7$ (distances defined in Supplementary Figure 9 inset). Using the values of $\xi_{ab}(T)$ and J_c determined from neutron scattering [7], we find that $\xi_{ab}(T)$ reaches d_{2NN} at T ~ 65 K (Supplementary Figure 9). Plugging $N(65\ K) = 7$ into the mean field equation gives a temperature of ~ 75 K, which is reasonably close to 65 K. Thus we estimate the mean field condition to be satisfied at T_{3D} = 70 K, in reasonable agreement with our observed value of T_{3D} ~ 60 K.

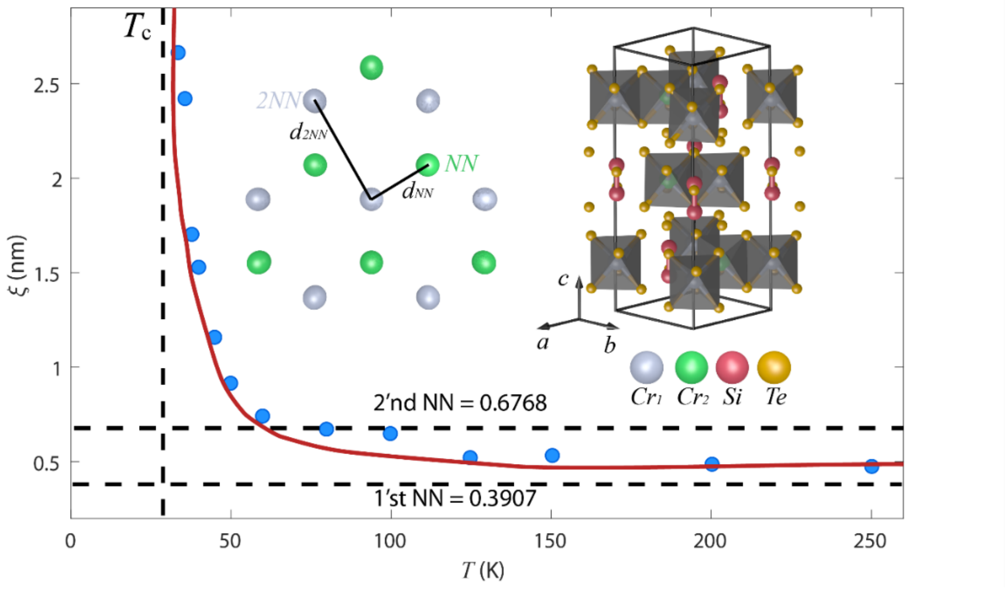

Supplementary Figure 9. 2D Coherence length as function of temperature [digitized from Ref. [7] Fig. 2(b)]. The blue dots are data points and the red line is a guide to the eye. Vertical dashed line marks T_c and the horizontal dashed lines mark the 1'st and 2'nd nearest neighbors distances. Inset (right) is an illustration of the crystal structure highlighting the two inequivalent Cr sites in silver and green. Inset (left) is an illustration of the honeycomb structure of the Cr atoms in a single layer. The two inequivalent sites occupy the two honeycomb structure sub-lattices. Each Cr atom has 3 nearest neighbors (NN) and 6 next nearest neighbors (2NN).

Supplementary Note 9. Bond model results for a rank-2 tensor response

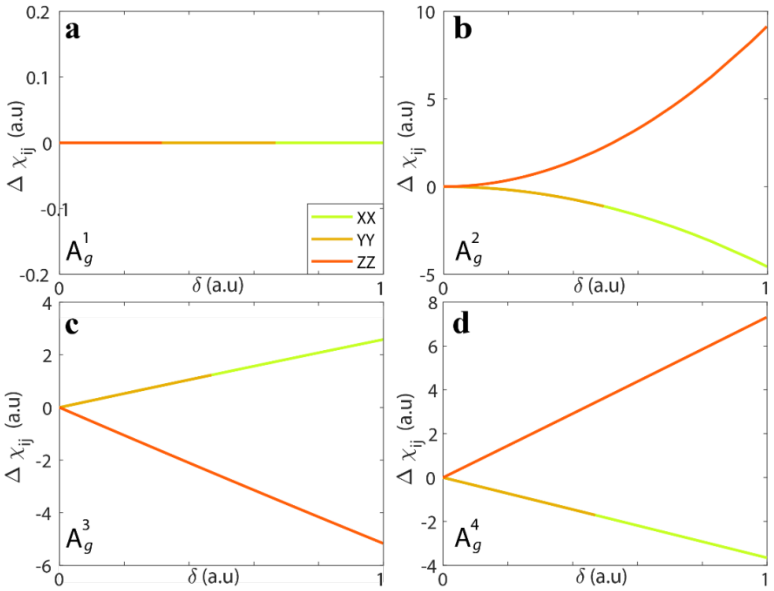

Supplementary Figure 10. Changes to a second rank polar tensor (reduced under point group $\bar{3}$) induced by totally symmetric distortions along the 4 normal coordinates described in the main text.

To understand how totally symmetric distortions along the 4 normal coordinates $A_g^1 \rightarrow A_g^4$ affect a rank-2 polar tensor χ_{ij} rather than a rank-4 polar tensor χ_{ijkl} as studied in the main text, we performed analogous hyper-polarizable bond model calculations for χ_{ij}. For a crystal with $\bar{3}$ point group symmetry, there are 3 non-zero elements in χ_{ij}, of which only 2 are independent (*xx* = *yy*, *zz*). The hyper-polarizable bond model calculations in Supplementary Figure 10 show that for the A_g^1 distortion, no elements change whereas for the other 3 distortions, all elements change. This shows that a rank-2 tensor response does not have enough degrees of freedom to differentiate between the distortions and one must appeal to a higher-rank nonlinear response.

We note that even though techniques such as Raman, IR or time-resolved optical spectroscopy are “mode specific”, they are sensitive to phonon modes and not to static distortions like shown in our work. While it is reasonable to assume that a static distortion in some basis function will have an effect on the corresponding phonon (e.g. a change in its amplitude, frequency or lifetime), such effects commonly arise from non-linear couplings between the modes, or between the modes and other crystal degrees of freedom through for example spin-phonon or electron phonon coupling. This is in fact the reason for the changes at T_{3D} shown in the inset of Figure 3b. Therefore the interpretation of such measurements (via frequency, amplitude or lifetime) cannot be associated directly to a static change in the corresponding basis function. A clear example can be taken from $CrSiTe_3$, where a change in the frequency of 3 IR modes, which lack a center of inversion, was reported in Ref. [20] to occur close to T_c, even though the crystal obviously remains centrosymmetric.
