# Supplementary material for: Dimensional crossover in a layered ferromagnet detected by spin correlation driven distortions
Source: Nat Commun. 2019 Apr 10;10:1654. doi: 10.1038/s41467-019-09663-3 (PMC6458139; doi:10.1038/s41467-019-09663-3)
Supplement: Supplementary file 2 — Peer Review [file 41467_2019_9663_MOESM2_ESM.docx]

**Editorial Note:** This manuscript has been previously reviewed at another journal that is not operating a transparent peer review scheme. This document only contains reviewer comments and rebuttal letters for versions considered at Nature Communications.
